# The relationships between teachers’ emotional health and stress coping

**DOI:** 10.3389/fpsyg.2023.1276431

**Published:** 2023-11-20

**Authors:** Arūnas Emeljanovas, Stanislav Sabaliauskas, Brigita Mežienė, Natalja Istomina

**Affiliations:** Faculty of Medicine, Vilnius University, Vilnius, Lithuania

**Keywords:** teachers, emotional health, well-being, psychological distress, burnout, stress coping

## Abstract

**Introduction:**

Teaching is a profession that involves challenges to emotional health. Teachers experience high levels of work-related stress, which causes symptoms such as anxiety, depression and burnout. Teachers’ mental health affects not only their own well-being, but also the quality of education and student achievement. Coping strategies can effectively improve teachers’ emotional health. The aim of this study is to assess the relationship between teachers’ emotional health and stress coping.

**Methods:**

The sample consisted of 385 teachers from Lithuania, with an average age of 50.2 (±9.62) years and 24.9 (±11.8) years of school experience. The WHO-5, the Kessler Psychological Distress Scale (K6), the Spanish Burnout Inventory, Educational Version (SBI-Ed) and the Coping Strategies Scale were used in the study.

**Results:**

The study results show that teachers’ age, seniority, size of residence, and marital status do not affect their emotional health, but their financial situation and hobbies have a positive impact on enthusiasm. Effective coping strategies such as problem solving, exercise, and hobbies improve emotional well-being, whereas negative coping methods such as self-isolation and alcohol consumption lead to psychological distress and lower enthusiasm at work.

## Introduction

Teaching is a socially valuable, meaningful and influential profession. However, rapid social and technological changes and constant monitoring of society are increasingly challenging for teachers (Carroll et al., 2022). Due to the emerging challenges and uncertainties of the social world and professional complexity, including collegial competition, standardized performance, increasing accountability, and rapid assessment, the teaching profession is considered to be at high risk (Zhang et al., 2023). Teaching has been associated with increased stress (Carroll et al., 2022; Li et al., 2022). High teacher stress is associated with physical and mental health problems, and can have negative occupational consequences. The latter are associated with professional burnout and turnover of qualified teachers (Puhakka et al., 2021; Gearhart et al., 2022). Therefore, teachers’ well-being is not only important for teachers and the school community but also for future society (McCallum et al., 2017; Yu et al., 2022).

Although empirical evidence is still scarce, new research results suggest that teachers’ well-being impacts their relationships with students, school climate, and student achievement (López et al., 2017). Thus, empirical findings suggest that teachers’ psychological states affect not only their own personal well-being but also their well-being, and that students’ mental health and well-being are related to teachers’ well-being (Harding et al., 2019). Research also shows that teacher well-being is critical to young people’s potential for innovation and productivity (McCallum, 2021), is related to teaching effectiveness, and influences students’ academic achievement (Harding et al., 2019; Hascher and Waber, 2021). In addition, teacher well-being impacts educational management (Hascher and Waber, 2021), and interpersonal relationships within the teaching team and students’ academic achievement are significantly interrelated (Göktaş and Metin, 2022).

While several studies have demonstrated the importance of teacher well-being, previous research has focused on the ‘dark side’ of teacher well-being (Li et al., 2022). This research highlighted that teaching is a highly demanding, anxiety-provoking, and stressful profession that can lead to job dissatisfaction, mental illness, and impaired well-being. However, ideas from positive psychology (Seligman and Peterson, 2003) have led researchers to focus more specifically on well-being in education. At present, there is growing interest and concern among researchers, managers, and policymakers about the well-being of teachers, not only to find ways to reduce the risk of stress, exhaustion, and burnout but also to help teachers flourish – to increase their optimism and self-efficacy.

### Teachers’ well-being

Well-being is determined by several interacting social, psychological, and biological factors as well as general levels of health and illness (Klapp et al., 2023). Indeed, ‘well-being’ is part of the concept of health and is related to physical, mental, and social health. The World Health Organization (WHO) notes that well-being is *“...not merely the absence of disease or infirmity”* and that mental health is *“...a state of well-being in which every individual realizes his or her own potential, can cope with the normal stresses of life, can work productively and fruitfully, and is able to make a contribution to her or his community”* (WHO, 2014). The understanding of the WHO definition of well-being is meaningfully expanded by the salutogenic perspective on health, which defines health as *“the process by which individuals maintain their sense of coherence and ability to function in the face of changes in themselves and their relationships with their environment”* (Antonovsky, 1987).

Thus, well-being must be understood in a broader sense than in the absence of illness. Rather, it refers to the healthy and successful functioning of teachers in their work and the ability of teachers to create a positive balance between the available resources and the challenges and demands (psychological, physical, individual, social, and environmental) that arise (Benevene et al., 2020). Well-being encompasses cognitive and affective as well as physical and mental components, and dispositional, personal, organizational, and environmental factors (Benevene et al., 2020). Well-being can be conceptualized and assessed as (1) objective well-being, which reflects what others can objectively measure and observe, such as economic resources, political circumstances, physical health status, number of social connections, and literacy, and (2) subjective well-being, which reflects individuals’ subjective experiences, such as happiness, emotions, engagement, life satisfaction, quality of social relationships, competencies, and achievements (Forgeard et al., 2011).

Research shows that teachers’ well-being is related to many aspects, such as personal characteristics and job engagement (Jelińska and Paradowski, 2021), workload or students’ classroom behavior (Chan et al., 2021), positive relationships with students, colleagues and families, as well as students’ better academic performance (Baumeister et al., 2003), emotion regulation, a positive work environment and teachers’ self-efficacy (feeling successful as a teacher) (Collie et al., 2015; Sohail et al., 2023); and contextual factors such as institutional resources and the amount of support received (Kumpikaitė-Valiūnienė et al., 2021). Negative work environments and negative emotions, as well as feelings of being ostracized or bullied by colleagues, are factors in teacher burnout (Sohail et al., 2023). Thus, teachers’ well-being reflects their professional satisfaction and happiness, and is perceived as a multidimensional and multilayered psychological construct (Li et al., 2022). It has been associated with other phenomena, including positive emotions and satisfaction, motivation and commitment, resilience and thriving, and negative associations with teacher stress and burnout (Burić et al., 2019; Hascher and Waber, 2021).

### Psychological distress and burnout

Psychological distress refers to non-specific symptoms of stress, anxiety and depression (Viertiö et al., 2021). Psychological distress manifests itself in a series of mental disorders such as anxiety, sadness, irritability, reduced self-confidence, and emotional distress, and is closely linked to physical and mental illnesses and poorer quality of life (Ozoemena et al., 2021). Distress is also associated with worthlessness, hopelessness, helplessness (Kessler et al., 2002), and burnout (Näring et al., 2011; Chen et al., 2022).

Burnout is defined as a reaction to prolonged stress at work, which results from a sustained mismatch between an employee’s resources and the demands placed on the employee (Bakker and Demerouti, 2017). Burnout is related to the work environment and motivational factors (Fernet et al., 2012), and manifests itself with different physical and mental health impairments. Burnout syndrome manifests itself as fatigue, characterized by loss of motivation, lack of energy, and apathy resulting from the persistent stress experienced in challenging work situations (Gabriel and Aguinis, 2022). Burnout is associated with reduced personal satisfaction and depersonalization (Bianchi et al., 2015), high levels of distress and multiple physical and mental problems, sleep disturbances, reduced productivity and motivation, and an increased risk of sickness absence (Stier-Jarmer et al., 2016). Burnout is characterized by feelings of physical, emotional, and mental exhaustion, manifested by emotional strain, negative attitudes toward work-life balance (Schilling et al., 2018), indolence, and guilt (Figueiredo-Ferraz et al., 2021).

Emotional exhaustion is a key symptom of burnout (Arens and Morin, 2016). People who are burnt out are emotionally exhausted and feel negative and disengaged from their work, which leads to lower productivity, stifled creativity and innovation, work accidents, absenteeism, and physical and mental illnesses. Studies in the field of education have shown that teachers’ enthusiasm drops sharply when burnout increases (Voss et al., 2023). Teachers’ emotional exhaustion leads to less engagement and effort in lesson planning, as well as more negative attitudes toward students (Frenzel et al., 2021).

### The present study

Compared to other professions, teachers are at high-risk of different mental health issues. While stress is a normal response to distressing or threatening events, it becomes pathological when chronic (Seo et al., 2017). A study on work-related stress in 26 professions (Johnson et al., 2005) found that teachers had one of the lowest levels of psychological well-being among all professions studied. Teachers around the world still face problems of stress and burnout, which cause anxiety and depression (Agyapong et al., 2022).

Educators consistently report higher levels of behavioral, psychological, and physiological symptomatology due to work-related stress (Ormiston et al., 2022). Changing working conditions, social acceleration, digitalization, and working with children with special needs place increasing demands on teachers. Consequently, the difficulties and challenges experienced reduce job satisfaction and commitment, cause tension and anxiety, and may lead to depression or burnout. These symptoms are risk factors of poor physical and mental health.

Stress management strategies can reduce stress and its consequences, and improve mental health. Although the mental health of teachers is becoming an increasingly important issue, there is a lack of research on the use of stress coping strategies in this area. To address this societal issue, it is important to empirically assess the impact of coping strategies on teachers’ mental health. Therefore, our study adopted a multidimensional approach that included a range of factors related to teachers’ mental well-being, symptoms of stress and burnout, and ways of coping with them. The aim of this study is to assess the relationship between teachers’ emotional health and stress coping.

## Materials and methods

### Participants

A cross-sectional study of teaching staff based on cluster (area) random sampling. The study involved 385 teaching staff (90.9% of whom were women), representing all regions of the country (Table 1). Teachers from 56 schools were invited to take part in the study. The study sample was selected across all 10 regions of Lithuania. Two thirds of the participants (65.6%) represented metropolitan schools, the rest represented small towns and rural areas. The average age of the participants was 50.2 (±9.62) years, the youngest participant was 25 years old and the oldest was 69 years old. The average number of years of schooling was 24.9 (±11.8) years.

**Table 1:** Baseline characteristics of the participants (*n* = 385).

Characteristics	Indicators
Age (years)	50.2 (SD = 9.62)
Work experience in school (years)	24.9 (SD = 11.8)
Teachers gender	
% Females	90.9 (*n* = 349)
% Males	9.1 (*n* = 35)
Marital status	
%Single	20.1 (*n* = 77)
%Married or have a life partner	79.9 (*n* = 308)
Settlement areas	
City (more than 200,000 inhabitants)	30.1 (*n* = 116)
City (50–200,000 inhabitants)	24.7 (*n* = 95)
Small town (<50,000 inhabitants)	29.1 (*n* = 112)
Rural	15.8 (*n* = 61)
Teachers’ specialization	
% Subject teachers	62.2 (*n* = 240)
% Primary school teachers	14.6 (*n* = 56)
% Teachers engaged in other pedagogical activities	14.8 (*n* = 57)
% Social pedagogues	5.2 (*n* = 20)
% Teachers’ assistants	1.8 (*n* = 7)
% Speech therapists	1.3 (*n* = 5)

### Study design and procedure

This is a cross-sectional study. Nested random sampling was applied. At least one school from each county was selected in all 10 counties of Lithuania. The researchers received permission from the school administration to collect data. The researchers provided the school administration with links to the online questionnaires and informed consent. School administrators shared links to the questionnaires with teachers through internal communication channels. The study was carried out in accordance with the Declaration of Helsinki and the protocol was approved by the Ethics Committee of the Department of Nursing, Vilnius University, Faculty of Medicine ((1.3) 150000-KT-214). All teachers who agreed to participate in each selected school became a participant. Filling out the questionnaire took about 30 min. Data collection took place between February and April 2023.

### Measures

#### Survey

A questionnaire was developed for the study, which consisted of three parts:(1) a guide for participants and informed consent, (2) socio-demographic variables, and (3) variables describing teachers’ psychological well-being, psychological distress and signs of burnout, and the psychological environment at work. The inventories were selected on the basis of their suitability to the aims of the study and on the basis of their well-established psychometric properties.

#### Sociodemographic data

All respondents were given a standard set of sociodemographic variables (e.g., age, gender, occupation, social status, and perceived income). Information on the respondents also includes data on the size of the settlement and the size of the school where they work.

***Psychological well-being*** was assessed using the WHO-5 scale (Cronbach’s α - 0.888), which measures how often a person is active, energetic, rested, relaxed, has hobbies, and is in a good mood, with responses on a Likert scale ranging from 0 - “never” to 5 - “all the time” (WHO, 1998). The WHO-5 scale has been translated into more than 30 languages and is used in research projects around the world to assess general well-being in a wide range of research fields (Topp et al., 2015).

WHO-5 index score, which is the sum of 5 items multiplied by 4 (ranging from 0 to 100), was used for statistical analysis. Good well-being is defined as having an index score between 51 and 100; poor well-being is defined as having an index score between 29 and 50; and the risk of depression is defined as having an index score of 28 or less. The dependent variable (WHO-5 well-being index) was transformed into three categories and coded as follows: 1 – “good” 2—“poor,” 3—“depression risk.”

***Teachers’ psychological distress*** was assessed using the Kessler Psychological Distress Scale (K6; Cronbach’s α - 0.877) (Kessler et al., 2002). The scale contains 6 questions that reflect the frequency with which symptoms of anxiety or stress have been experienced in the last 4 weeks. The responses were scored on a 5-point scale, ranging from ‘all the time’ (score 0) to ‘never’ (score 4) and afterwards were rescored in order the higher total score to indicate greater psychological distress.

***Teachers’ burnout*** symptoms were investigated using the Spanish Burnout Inventory, educational version (SBI-Ed; Cronbach’s α - 0.877), which is specifically adapted to the field of education (Gil-Monte et al., 2010). The scale consists of consists of 20 items, which are divided into four subscales: (1) *enthusiasm for work* (5 items) - the individual’s desire to achieve goals at work as a source of personal pleasure; (2) *psychological exhaustion* (5 items) - the manifestation of emotional and physical exhaustion due to the daily interaction with problematic people at work; (3) *indolence* (6 items) - the emergence of negative attitudes and indifference to the individual; and (4) *guilt* (4 items), which arise from the emergence of negative attitudes and attitudes toward others. Items were scored on a five-point Likert-type scale ranging from 0 (‘never’) to 4 (‘very often: every day’).

***Coping strategies*** were assessed by analyzing participants’ behavior when they felt depressed, low in mood, anxious, or stressed, including communication with family and friends, contact with mental health professionals, prayer, physical activity and hobbies, harmful habits (e.g., smoking, alcohol, etc.), problem-solving and ignoring, feelings of guilt (blaming oneself, others, and fate), and self-isolation. Respondents’ answers were marked on a scale ranging from “never” to “almost always.”

### Statistical analysis

A descriptive statistical analysis was performed for all study variables, as well as correlation and mean comparison analyses. The distribution of variables in each group was calculated using frequency-distribution tests. The missing data ranged from 0.6 to 1.5 percent for different variables. Due to that small proportion, imputation procedure was excluded, instead those few participants were excluded from the certain calculations where those variables were included. The relationships between the variables were calculated using Spearman’s correlations. Hierarchical regression analysis was used to explain the relationship between one scale dependent variable and independent variables. Enter method was used. The internal consistency of the scales was assessed using Cronbach’s alpha (>0.7). All analyses were performed using the statistical package SPSS 24.0 (SPSS Inc., Chicago, IL, USA).

## Results

The findings indicated that the mental health or psychosomatic symptoms of teachers were not associated with their teaching experience or their duration of service in the workplace (*p* > 0.05). Teachers’ work location in cities or suburbs of different sizes, as well as their marital status, did not indicate a relationship between teachers’ mental health or psychosomatic symptoms (*p* > 0.05) (Table 2). More than half (56.9%) of the teaching staff had good psychological well-being, almost a third (31.1%) had moderate well-being, and 12% of the teaching staff were at risk of depression. Almost one-third of the teachers (32.2%) showed signs of high psychological distress. It was found that teachers’ enthusiasm was above average (3.7 ± 0.74), indolence at work (1.99 ± 0.63), and guilt at work (1.89 ± 0.54) were at low levels. However, teachers’ psychological exhaustion was quite high, with a mean score of (2.81 ± 0.83) in the sample.

**Table 2:** Indicators of teachers’ mental health and the psychological climate of their working environment.

Phenomena	Indicators	Mean (SD) or % (*n*)
Psychological well-being	Good well-being	56.6%
	Poor well-being	31.1%
	Depression risk	12.0%
Psychological distress	High psychological distress	32.2%
	Low psychological distress	67.8%
Burnout	Enthusiasm for work	3.71 (0.74)
	Psychological exhaustion	2.81 (0.83)
	Indolence	1.99 (0.63)
	Guilt	1.89 (0.54)

**Figure 1:**
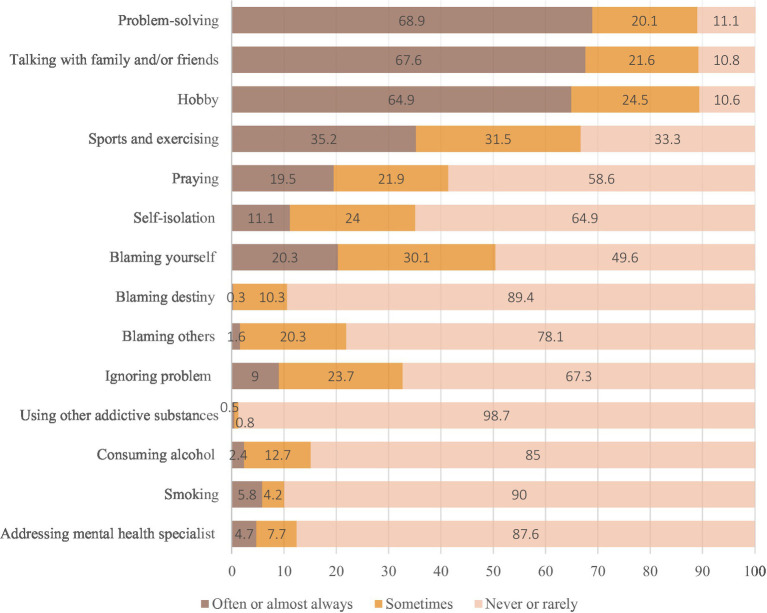
Teachers’ cooping strategies.

The analysis of coping strategies showed that when teachers felt depressed, low in mood, anxious, or stressed, the majority of teachers tended to use problem-solving (68.9%), talk with family and/or friends (67.6%), and engage in hobbies (64.9%), and that 20–25% of the participants sometimes used these strategies. 35.2% participated in sports, and 31.5% sometimes participated in sports (Figure 1).

Meanwhile, 9% of the respondents ignored these problems. Some respondents noted that they drank alcohol (2.4%), smoked (5.8%), or took other addictive substances (0.5%). 10–20% tend to blame themselves, 11.1% of teachers tended to isolate themselves (24.0% did this sometimes), and 20.3% blamed themselves (30.1% did this sometimes). Only a small proportion of teachers (4.7%) self-referred to mental health specialists.

The correlation analysis revealed that teachers’ psychological well-being and enthusiasm for work are positively correlated with sports and exercising (*r* = 0.103–0.216; *p* < 0.001), hobby (*r* = 0.250–0.324, *p* < 0.001), and problem-solving (*r* = 0.252–0.268; *p* < 0.001). Meanwhile, teachers’ psychological distress, psychological exhaustion, indolence, and guilt were associated with ignoring problems (*r* = 0.152–0.297; *p* < 0.001), doing less sports (*r* = −0.145–0.195; *p* < 0.001), and less time spent on hobbies (*r* = −0.135–0.219; *p* < 0.001). In addition, teachers’ psychological exhaustion, indolence, and guilt were positively related to alcohol consumption and other addictive substances (*r* = 0.147–0.241; *p* < 0.001) and ignoring problems (*r* = 0.152–0.297; p < 0.001). Teachers’ self-isolation, blaming others, blaming destiny, and blaming themselves were also significantly negatively related to teachers’ well-being, psychological distress, and enthusiasm for work (Table 3).

**Table 3:** Bivariate correlations among study variables.

Indicators	Psychological well-being	Psychological distress	Enthusiasm for work	Psychological exhaustion	Indolence	Guilt
Talking with family and/or friends	0.093	−0.123^*^	0.157^**^	−0.077	−0.131^*^	−0.147^**^
Addressing mental health specialist	−0.053	0.085	0.031	−0.021	0.017	0.081
Sports and exercising	0.216^**^	−0.195^**^	0.103^*^	−0.145^**^	−0.094	−0.179^**^
Hobby	0.324^**^	−0.305^**^	0.250^**^	−0.219^**^	−0.135^**^	−0.188^**^
Problem-solving	0.252^**^	−0.301^**^	0.268^**^	−0.201^**^	−0.138^**^	−0.209^**^
Praying	−0.052	0.053	0.078	−0.034	−0.006	−0.001
Smoking	−0.026	0.023	0.011	0.019	0.035	0.119^*^
Consuming alcohol	−0.063	0.087	−0.109^*^	0.147^**^	0.212^**^	0.241^**^
Using other addictive substances	−0.029	0.091	−0.103^*^	0.047	0.189^**^	0.214^**^
Ignoring problem	−0.115^*^	0.170^**^	−0.195^**^	0.152^**^	0.297^**^	0.263^**^
Blaming others	−0.318^**^	0.285^**^	−0.285^**^	0.345^**^	0.395^**^	0.374^**^
Blaming destiny	−0.242^**^	0.304^**^	−0.219^**^	0.242^**^	0.271^**^	0.348^**^
Blaming yourself	−0.289^**^	0.350^**^	−0.200^**^	0.347^**^	0.225^**^	0.332^**^
Self-isolation	−0.309^**^	0.395^**^	−0.294^**^	0.266^**^	0.260^**^	0.296^**^

* *p* < 0.05; ** *p* < 0.01.

Hierarchical regression analysis was performed. Model 1 in each regression included sociodemographic variables. Model 1 indicated the percentage of variance they covered and revealed the unique importance of sociodemographic variables for psychological health. They also served as covariates in Model 2 which disclose the controlled for covariates relationships of stress coping strategies with psychological health variables. Results presented in Table 4 indicate that sociodemographic indicators in Model 1 predict only 3.3 percent of the variance of enthusiasm at work. Among them only financial status was significant – the higher the financial status in the family the more enthusiasm the schoolteachers experience at work. The financial status remained significant in Model 2. Among stress coping strategies in Model 2 engaging in a hobby and problem solving were positively related to higher enthusiasm at work, while blaming others and self-isolation predicted lower work-related enthusiasm. Altogether stress coping indicators explained 21.6 percent of the variance of enthusiasm.

**Table 4:** Sociodemographic and stress coping factors of enthusiasm at work, psychological exhaustion, inactivity and guilt (Std β).

Indicators	Psychological well-being		Psychological distress		Enthusiasm at work		Psychological exhaustion		Indolence		Guilt	
	Model 1	Model 2	Model 1	Model 2	Model 1	Model 2	Model 1	Model 2	Model 1	Model 2	Model 1	Model 2
Gender (women)	−0.161**	−0.143**	0.056	0.031	0.025	−0.018	0.081	0.088	−0.108*	−0.083	−0.035	−0.029
Age	−0.021	−0.008	0.005	−0.017	0.026	0.043	−0.031	−0.069	0.064	0.041	0.034	0.003
Work experience in years	0.058	0.007	−0.105*	−0.055	−0.042	−0.073	0.024	0.062	0.032	0.062	0.057	0.099*
Family status (in a relationship)	−0.041	−0.038	0.006	−0.010	−0.038	−0.035	0.097	0.077	0.044	0.054	−0.017	−0.015
Financial status	0.184***	0.185***	−0.212***	−0.199***	0.173^***^	0.199^***^	−0.129^*^	−0.144^**^	0.008	−0.019	0.069	0.055
Talking with family and/or friends		0.003		0.023		0.038		−0.014		−0.076		−0.048
Addressing mental health specialist		−0.013		0.029		0.044		−0.055		0.023		0.081
Sports and exercising		0.064		−0.047		−0.014		−0.024		−0.059		−0.096
Hobby		0.196***		−0.144**		0.136^*^		−0.109^*^		−0.003		−0.009
Problem-solving		0.111*		−0.176***		0.123^*^		−0.073		0.020		−0.069
Praying		−0.032		0.051		0.069		−0.039		0.025		0.017
Smoking		0.006		−0.022		0.095		−0.016		−0.039		0.032
Consuming alcohol		−0.015		0.035		−0.084		0.109^*^		0.127^*^		0.127^**^
Using other addictive substances		−0.001		0.051		−0.078		0.011		0.082		0.096*
Ignoring problem		0.020		−0.002		−0.055		0.009		0.181^***^		0.094
Blaming others		−0.193***		0.069		−0.175^***^		0.202^***^		0.271^***^		0.172^***^
Blaming destiny		0.000		0.091		0.025		0.001		0.031		0.123^*^
Blaming oneself		−0.119*		0.177**		−0.042		0.222^***^		0.029		0.156^**^
Self-isolation		−0.170**		0.227**		−0.174^***^		0.115^*^		0.123^*^		0.097
ΔR^2^	0.067***	0.250***	0.057***	0.308***	0.033*	0.216^***^	0.034*	248***	0.019*	0.261***	0.011	0.313^***^

* *p* < 0.05; ** *p* < 0.01; *** *p* < 0.001.

Similar results were found when analyzing the prediction of sociodemographic variables for the prevention of psychological exhaustion - financial status and engaging in a hobby are also significant. Consuming alcohol, blaming others and oneself predict greater psychological exhaustion. Stress coping indicators explained 24.8 percent of the variance in psychological exhaustion.

Indolence was positively associated with male gender in Model 1, alcohol consumption, ignoring problems, blaming others, and self-isolation in Model 2. 26.1 percent of the variance in indolence was explained by stress coping indicators.

Alcohol consumption, use of other addictive substances, blaming others and oneself, as well as blaming fate, are predictors of guilt. The stress coping indicators in this model explain almost a third of the variance (31.3 percent).

## Discussion

The aim of this study was to assess the relationship between teachers’ emotional health and stress coping. Although previous studies have already analyzed the consequences of stress and burnout syndrome experienced by teachers, we have gone beyond the study of these negative effects to include factors that will allow us to predict the direction of improvement of teachers’ emotional health and thus contribute to the overall well-being of schools.

Some research suggests that teachers’ ability to cope with or remain in school in the face of high job uncertainty, job stress, policy changes, and career challenges is most difficult at the beginning of their careers when they are prone to burnout and leaving the profession (e.g., Schaefer, 2013). However, in the case of our study, teachers’ age did not affect the stress experienced by teachers or their well-being. Additionally, teachers’ mental health and psychosomatic symptoms were not related to the size of the city in which they worked, marital status, or length of time in the same job.

While most of the teachers in the study had good well-being and did not experience significant psychological exhaustion, some teachers were at risk of depression, and one-third of the study participants experienced psychological distress. However, despite these indicators, the teachers in this study had high levels of enthusiasm, moderate levels of psychological exhaustion, and relatively low levels of indolence and guilt. This study showed that teachers’ psychological exhaustion, sluggishness, guilt at work, and psychosomatic indicators were not related to their financial situation. However, the study showed that teachers’ financial situation and hobbies are related to their psychological well-being and enthusiasm at work. The results of our study are in line with other studies on the relationship between financial satisfaction and subjective well-being (Ngamaba et al., 2020), which showed that financial satisfaction is moderately and positively related to subjective well-being. The authors note that this relationship is higher and stronger in developing countries when multiple items rather than single-item measures are used. Thus, the results of our study imply that teachers’ financial well-being can positively affect their enthusiasm and reduce psychological exhaustion. However, financial status was not related to teacher burnout. This means that improving teachers’ financial status alone does not prevent negative mental health outcomes. It should be noted that improving teachers’ mental health is essential for improving their working conditions and increasing job satisfaction (Peng et al., 2022).

Teachers’ enthusiasm emerged as an important dimension of the study, and was not only influenced by the financial situation. Teachers’ psychological well-being and enthusiasm are related to social, emotional, and instrumental support as well as to the support of their manager, administration, and colleagues. Enthusiasm is described as constructive emotional satisfaction that includes interest (Kunter and Holzberger, 2014). The enthusiasm of each teacher is a key factor in effective teaching, determining teachers’ well-being and behavior, and students’ cognitive, emotional, and motivational outcomes (Burić and Moe, 2020). Demonstrating enthusiasm is indicative of an individual’s invigorating and energetic nature (Keller et al., 2016). It should be noted that teachers’ enthusiasm is important for their personal well-being, quality of teaching, and engagement of students in the teaching process, as well as for the constructive impact and inspiration of students (Keller et al., 2016). Additionally, enthusiasm is significant for an individual’s participation in career development (Hakanen et al., 2006).

The results of this study broaden the understanding of teachers’ emotional health by highlighting the link between well-being and stress management strategies. This study highlights a number of stress coping strategies that teachers use when faced with challenges, such as timely problem solving, sports and exercise, and hobbies. Interestingly, talking to family and friends was one of the most common strategies used by teachers. Correlation analysis showed that this coping strategy had an impact on teachers’ enthusiasm; however, it was not related to their well-being. It is likely that talking to relatives improves emotional well-being during the interview but does not affect emotional health. Thus, for emotional health problems, it is appropriate to seek professional advice from specialists who can accurately diagnose the problem and suggest appropriate treatment (Soklaridis et al., 2020). In addition, the study showed that teachers’ self-isolation was a significant predictor of emotional health problems. This suggests that avoidance of communication and social relationships can lead to emotional health problems.

The study did not find any association between addressing mental health professionals and teachers’ well-being, enthusiasm, psychological distress, enthusiasm for work, psychological exhaustion, indolence, or guilt. Although there is evidence that specialist support can be effective in addressing emotional health problems, it is likely that these findings were obtained because only a small minority of professionals in the study mentioned that they were addressing mental health specialists. In addition, some teachers showed signs of psychological distress, a symptom of emotional health problems, indicating the need to address their mental health issues.

While some teachers managed their stress independently, some participants noted that they had resorted to harmful behaviors such as smoking, drinking alcohol, or using other addictive substances. In addition, some teachers showed signs of psychological distress, which is a sign of emotional health problems. Given that teachers’ well-being influences teaching effectiveness and students’ mental well-being, safety, and academic performance (Harding et al., 2019; Frenzel et al., 2021; Granziera et al., 2023), emotional health support and support for teachers is a valuable way to both improve the well-being of the teachers themselves and contribute to better learning conditions for students. Supporting teachers’ well-being and fostering teacher-student relationships is an important part of creating a healthy school climate (López et al., 2017). Therefore, providing psychological support to teachers is an untapped opportunity to improve educational practices. This insight is important because it shifts the focus from simply addressing stressors and reducing burnout to strengthening teachers’ psychosocial resources. Organizational support and teacher well-being are closely linked (Viac and Fraser, 2020), and teacher well-being is important for the optimal functioning of schools and educational systems (Hascher and Waber, 2021).

The results of our study demonstrate the importance of addressing teachers’ personal well-being. Improving subjective well-being is a top priority for many governments around the world (Ngamaba et al., 2020); therefore, identifying the key factors that influence teachers’ well-being is crucial for government policymaking. The findings are important for education managers and policymakers when designing interventions to improve teachers’ emotional health and well-being and to address work-life balance issues through preventative leisure activities. To reduce teacher burnout and attrition and improve the effectiveness and quality of education, it is recommended that education policy makers and institutions implement measures to enhance teachers’ emotional health. It is important to give priority to measures that promote teachers’ enthusiasm and enjoyment of leisure time, improving the work-life balance. Developing a culture of psychological support in schools is crucial to ensuring that teachers can seek professional help when they face emotional difficulties. By prioritising teachers’ well-being, policy makers can have a positive impact on the whole education system. Supporting and enhancing teachers’ emotional health and creating a supportive working environment can increase teaching effectiveness, reduce teacher burnout and turnover, and improve student outcomes.

The study has its strengths and limitations. Given the nested sampling, the wide range of teachers were involved including teachers in urban and rural areas, also those teaching in primary and secondary schools. Although, male gender was underrepresented. Meanwhile in the country among all teachers, male teachers cover 15 percent (Eurostat, 2021) in comparison with 9 percent in our study. This study used recognized and validated measures of psychological well-being, psychological distress, and burnout to ensure the reliability and validity of the results. This provides an opportunity for scientific dialogue on teachers’ well-being at the international level. Although the study analyzed different coping strategies, it did not assess and analyze the actual impact and effectiveness of coping strategies in improving teachers’ mental health. Therefore, assessing the impact of specific coping strategies could be a future research perspective that provides more practical insights based on empirical research.

## Conclusion

The study shows that teachers are very enthusiastic about their work, and most feel good about themselves. However, one-third of the participants have high levels of psychological distress and 11.9 per cent show signs of depression. Teachers’ age, length of service in the same school, size of settlement, and marital status are not related to their emotional health. Teachers’ psychological exhaustion and psychosomatic indicators are not related to their financial situation, but their financial situation and hobbies are positively related to teachers’ enthusiasm.

The most common coping strategies used by teachers are problem solving, talking to family/friends, sports and exercise, hobbies, praying, and blaming themselves; however, only problem solving, sports and exercise, and hobbies are positively related to emotional health. Ignoring problems, self-isolation, blame, and alcohol use are significantly related to greater psychological distress, psychological exhaustion, indolence, and guilt and predict a decline in well-being and enthusiasm at work.

## Data availability statement

The raw data supporting the conclusions of this article will be made available by the authors, without undue reservation.

## Ethics statement

The studies involving humans were approved by Ethics Committee of the Department of Nursing, Vilnius University, Faculty of Medicine. The studies were conducted in accordance with the local legislation and institutional requirements. The participants provided their written informed consent to participate in this study.

## Author contributions

AE: Conceptualization, Data curation, Formal analysis, Funding acquisition, Methodology, Project administration, Resources, Supervision, Validation, Writing – review & editing, Investigation. SS: Formal analysis, Visualization, Writing – original draft, Investigation, Methodology, Writing – review & editing, Data curation, Validation. BM: Conceptualization, Data curation, Formal analysis, Investigation, Methodology, Resources, Software, Supervision, Validation, Writing – review & editing. NI: Conceptualization, Methodology, Supervision, Writing – review & editing, Funding acquisition, Resources, Validation.

## Appendix

Data for missing values

**Table 2.** Indicators of teachers’ mental health and the psychological climate of their working environment.

**Well-being:** WHO scale:

**Table:**

		Frequency	Percent	Valid Percent	Cumulative Percent
Valid	Depression risk	46	11,9	12,0	12,0
	Poor well-being	119	30,9	31,1	43,1
	Good well-being	218	56,6	56,9	100,0
	Total	383	99,5	100,0	
Missing	System	2	,5		
Total		385	100,0		

**Psychological distress:** Kessler Psychological Distress Scale (K6):

**Table:**

		Frequency	Percent	Valid Percent	Cumulative Percent
Valid	High psychological distress	122	31,7	32,2	32,2
	Low psychological distress	257	66,8	67,8	100,0
	Total	379	98,4	100,0	
Missing	System	6	1,6		
Total		385	100,0		
